# Modulation of Dkk-3 and claudin-5 as new therapeutic strategy in the treatment of meningiomas

**DOI:** 10.18632/oncotarget.20047

**Published:** 2017-08-07

**Authors:** Maria Caffo, Emanuela Esposito, Valeria Barresi, Gerardo Caruso, Salvatore M Cardali, Mariagrazia Rinaldi, Raffaella Mallamace, Michela Campolo, Giovanna Casili, Alfredo Conti, Antonino Germanò, Salvatore Cuzzocrea, Letteria Minutoli

**Affiliations:** ^1^ Department of Biomedical and Dental Sciences and Morphofunctional Imaging, Unit of Neurosurgery, University of Messina, Messina, Italy; ^2^ Department of Clinical and Experimental Medicine, University of Messina, Messina, Italy; ^3^ Department of Human Pathology, University of Messina, Messina, Italy; ^4^ Unit of Anesthesia, University of Messina, Messina, Italy; ^5^ Department of Chemical, Biological, Pharmaceutical and Environmental Sciences, University of Messina, Messina, Italy

**Keywords:** atypical meningioma, claudin-5, Dkk-3, meningioma, Wnt pathway

## Abstract

Meningiomas are the most common tumors of the central nervous system, where the incidence is around 25% of all primary brain tumors. The optimal treatment is represented by total resection accompanied by the removal of the dura mater and bone when infiltrated by the tumor. The histological grading is the most important prognostic factor in the outcome. However, recurrences do occur in a significant proportion (10–25%) of cases, representing the most relevant clinical complication. Molecular therapies are providing to give different opportunities in the development of new treatments. The Dickkopf-related family of proteins includes four secretory proteins. The expression of the REIC/Dkk-3 gene is down-regulated in many tumor cell lines and could contribute to the immunomodulatory properties of the tissue microenvironment. An important role in carcinogenesis is played by Dickkopf protein-related protein 3, which is involved in embryonic development through its interaction and modulation of the pathway of the Wnt signal transduction. The mutations of this pathway are of clinical importance, because they lead to the onset of several cancers, including brain tumors, being also involved in tumor angiogenesis. The claudin-5, is an integral membrane protein, which regulate the permeability of the blood-brain barrier. In various pathological processes, including inflammation, trauma and tumor, claudin 5 regulate the change in endothelial or epithelial permeability, therefore, modification in claudin-5 expression may play a role in malignant transformation. The aim of our study is to demonstrate the role of Dkk-3 and claudin-5 in the pathogenesis of meningiomas. A more correct identification of the role of these proteins might suggest interesting and new molecular targets for future therapeutic protocols.

## INTRODUCTION

Meningiomas are the most common primary intracranial tumors [1]. A large amount of meningiomas, according to WHO classification shows a benign clinical behavior (grade I), but up to 20% are classified as atypical (grade II) or anaplastic (grade III) [2]. However, WHO grade I meningiomas, generally defined as benign, show a recurrence rates in the range of 7–20% and an evident possibility of progression to higher grades [1]. Grade II and grade III meningiomas have an aggressive course, with a high rate of recurrence, and, especially in grade III, a higher frequency of local invasion. Nowadays, treatment strategies for recurring meningiomas are inadequate. Chemotherapy and hormonal therapies own a reduced role, while, radiation therapy or stereotactic radiosurgery are limited by radiation neurotoxicity, tumor size, and injury to adjacent vascular structures or cranial nerves [1].

The Wingless (Wnt) signaling pathway in human cancers shows a valid role in uncontrolled cell growth, and recent studies have indicated that this pathway also plays a relevant role in meningioma progression [3–4]. Wnt pathway is implicated in the development of the BBB through regulating expression of several proteins. This pathway participates actively in the integrity check of the BBB and its disorder may affect BBB permeability. Emerging evidence that Wnt pathway controls claudin-5 expression by preventing the nuclear accumulation of β-catenin, which repress the claudin-5 promoter, provides a crosstalk between these endothelial junctional structures. Claudin proteins represent a large family of integral membrane proteins crucial for TJ formation and function, and are abnormally regulated in several human cancers [7]. Claudin-5 is a key component of the TJ strand, particularly in brain endothelial cells. The major role of claudin5 is to decrease the permeability to ions. In various pathological processes, including inflammation, edema, toxic damage, trauma and tumor, claudin-5 regulate the change in endothelial or epithelial permeability [8]. Therefore, in the light of these observations, modification in claudin-5 expression may play a role in malignant transformation. Dickkopf protein-related protein 3 (Dkk-3), is one of the most promising tumor suppressor molecules able to modulate the Wnt pathway [5]. However, the physiological significance of altered Dkk-3 expression in cancer and its potential growth inhibitory outcome are unidentified. To date, the most evident and consistent anti-tumor effect of Dkk-3 is its inhibitory capacity on cancer cell growth [6].

The purpose of this study was to evaluate the expression of Dkk-3 and claudin-5 in a series of 30 WHO grade I and grade II meningiomas. Nowadays, no study has evaluated the expression of Dkk-3 and claudin-5 in meningiomas. Our findings suggest that Dkk-3 and claudin-5 could be considered as potential targets for therapeutic approaches in the treatment of meningiomas.

## RESULTS

### Clinical data

The first series include patients with meningioma grade I. This series includes 15 patients, 10 women and 5 men, aged between 46 and 77 years with an average age of 60.6%. Meningiomas were located in the frontal region (6 cases), parasagittal (5 cases), sphenoid ridge (2 cases), and in the posterior fossa (2 cases). Preoperative symptoms were headache in all patients, seizures (4 patients), and visual disturbance (2 patients). The neurological evaluation showed positive Babinski sign and hemiparesis (7 patients), and papilledema (2 patients). All patients undergone MRI study. MRI revealed extra-axial masses with signal characteristics of isointensity to slight hypointensity relative to grey matter on the T1-weighted sequence and isointensity to slight hyperintensity relative to grey matter on the T2 sequence. After contrast administration, meningiomas demonstrate intense, homogeneous enhancement; some cases, however, showed areas of central necrosis or calcification that do not enhance. On MRI, calcification may also be appreciated on T2-weighted sequences as areas of low signal intensity. All patients underwent surgical treatment. In 13 patients (Table 1), total surgical excision (Simpson grade I) was obtained (86,8%), while in the other two patients, a partial tumor excision (Simpson grade III and IV) was performed because of severe adhesions to vascular and eloquent nervous structures. The histological type and grade of the specimens were classified according to the recent WHO classification. In this series of meningiomas, 7 were fibrous, 6 were meningothelial, and 2 cases were transitional. Necrosis was histologically evidenced in the same cases with central areas of necrosis at imaging. However, histological criteria for grade II diagnosis were not fulfilled. The duration of follow-up ranged from 6 to 60 months. In this series, we observed no recurrence. One patient died from a disease not related to meningioma. The cases characterized by a partial removal of the lesion are under careful clinical and neuroradiological observation.

**Table 1:** Correlation between grade of tumor resection (Simpson's scale) and recurrence

Simpson's Scale	Grade I meningiomas	Recurrence	Grade II meningiomas	Recurrence
**Grade 1**	**13**	/	**12**	/
**Grade 2**	/	/	/	/
**Grade 3**	**1**	/	**1**	/
**Grade 4**	**1**	/	**2**	**2**
**Grade 5**	/	/	/	/

The second series comprises patients affected by WHO grade II meningioma. This series included 11 women and 4 men, aged between 50 and 76 years with an average age of 62.5%. Meningiomas were located in parasagittal area (6 cases), in the frontal lobe (4 cases), in the left fronto-temporal region (2 cases), in the sellar region (2 cases), and sphenoid ridge (1 case). Initial symptoms were headache in all patients, seizures (6 patients), and visual deficits (3 patients). Two patients showed psychological changes, characterized by frontal syndrome. Neurological examination documented positive Babinski sign and hemiparesis (9 patients), and papilledema (3 patients). All patients undergone MRI study. Also in this series, the patients underwent surgical treatment. In 12 patients (80%), total surgical excision (Simpson grade I) was obtained, while in the remaining three patients, a partial tumor excision (Simpson grade III and IV) was performed. In these cases we observed a clear invasion of the venous sinuses (2 cases), and a tough adhesions to important vascular structures (3 cases). All the meningiomas were classified, according WHO scale, as atypical meningiomas (grade II). None of the cases showed histological evidence of brain infiltration. The duration of follow-up ranged from 6 to 60 months. In this series, recurrences were documented by neuroradiologic studies performed during this period. The entire recurrence rate for cases with subtotal resection was 20% (Table 1). Two patient died from a disease not related to meningioma. The cases characterized by a partial removal of the lesion are under careful clinical and neuroradiological observation. One patient died due to brain edema related to the recurrence.

### Immunohistochemistry and western blot analysis

**Figure 1:**
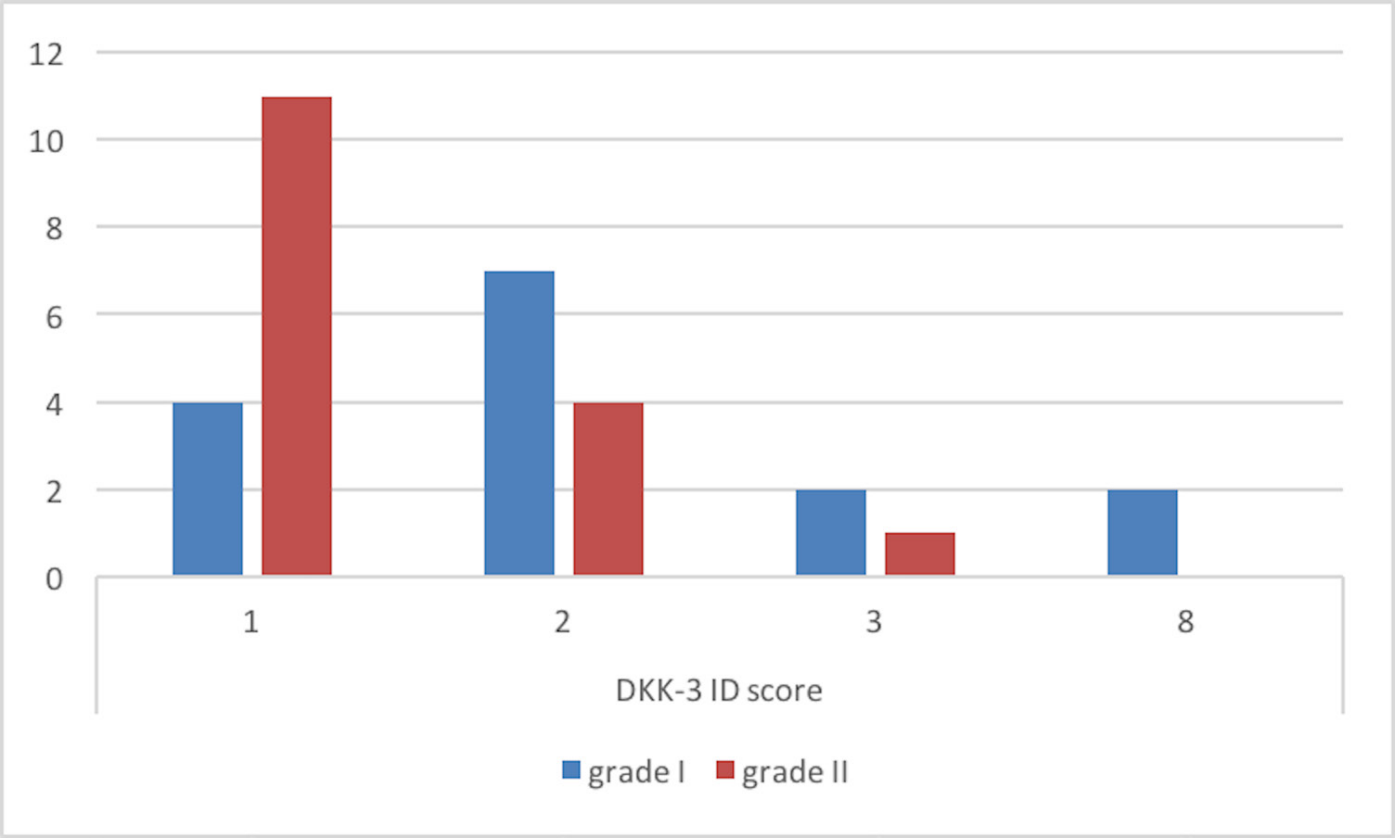
Dkk-3 immuno-expression in meningiomas

**Figure 2:**
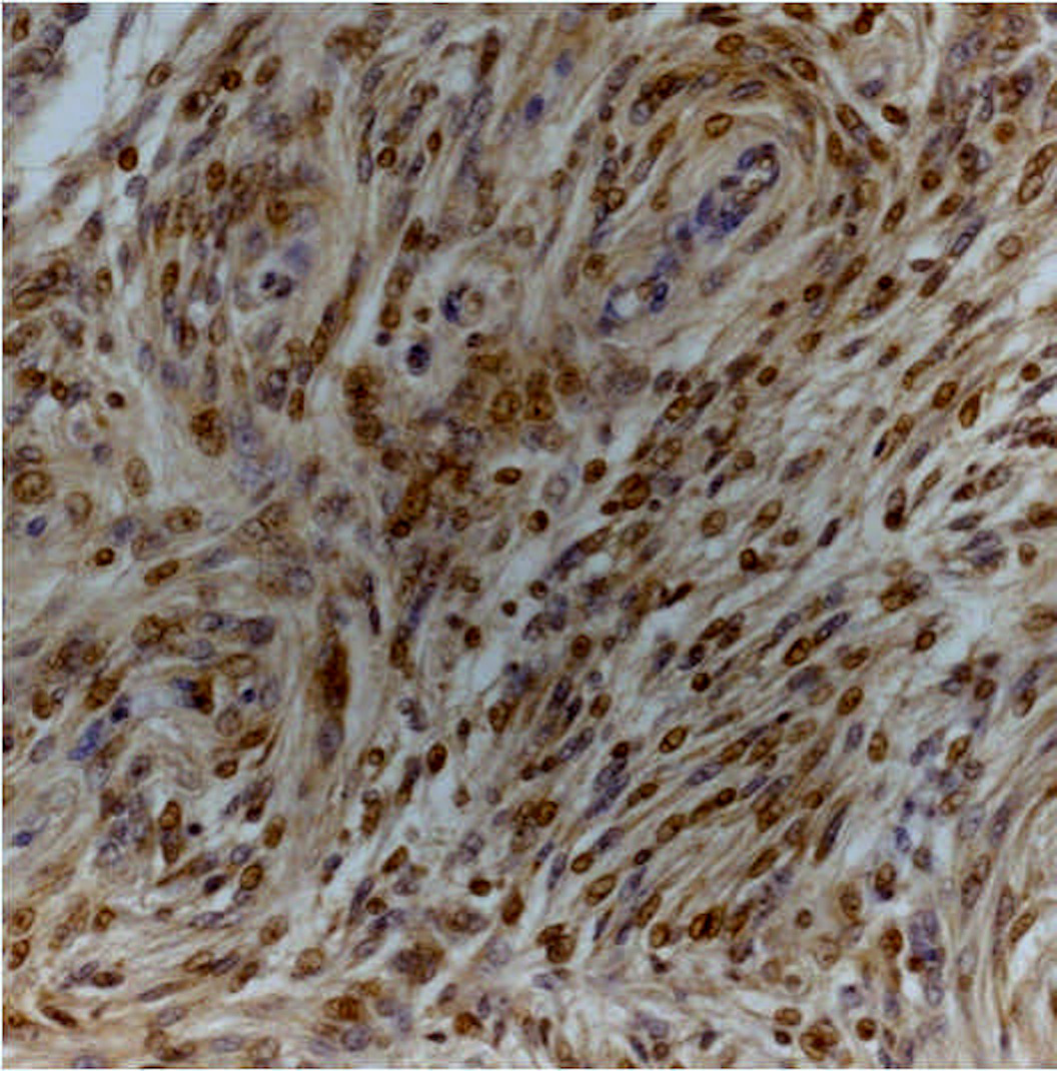
Transitional (grade I) meningioma showing moderately intense cytoplasmic and nuclear staining for Dkk3 (Dkk3 stain; original magnification, x200)

**Figure 3:**
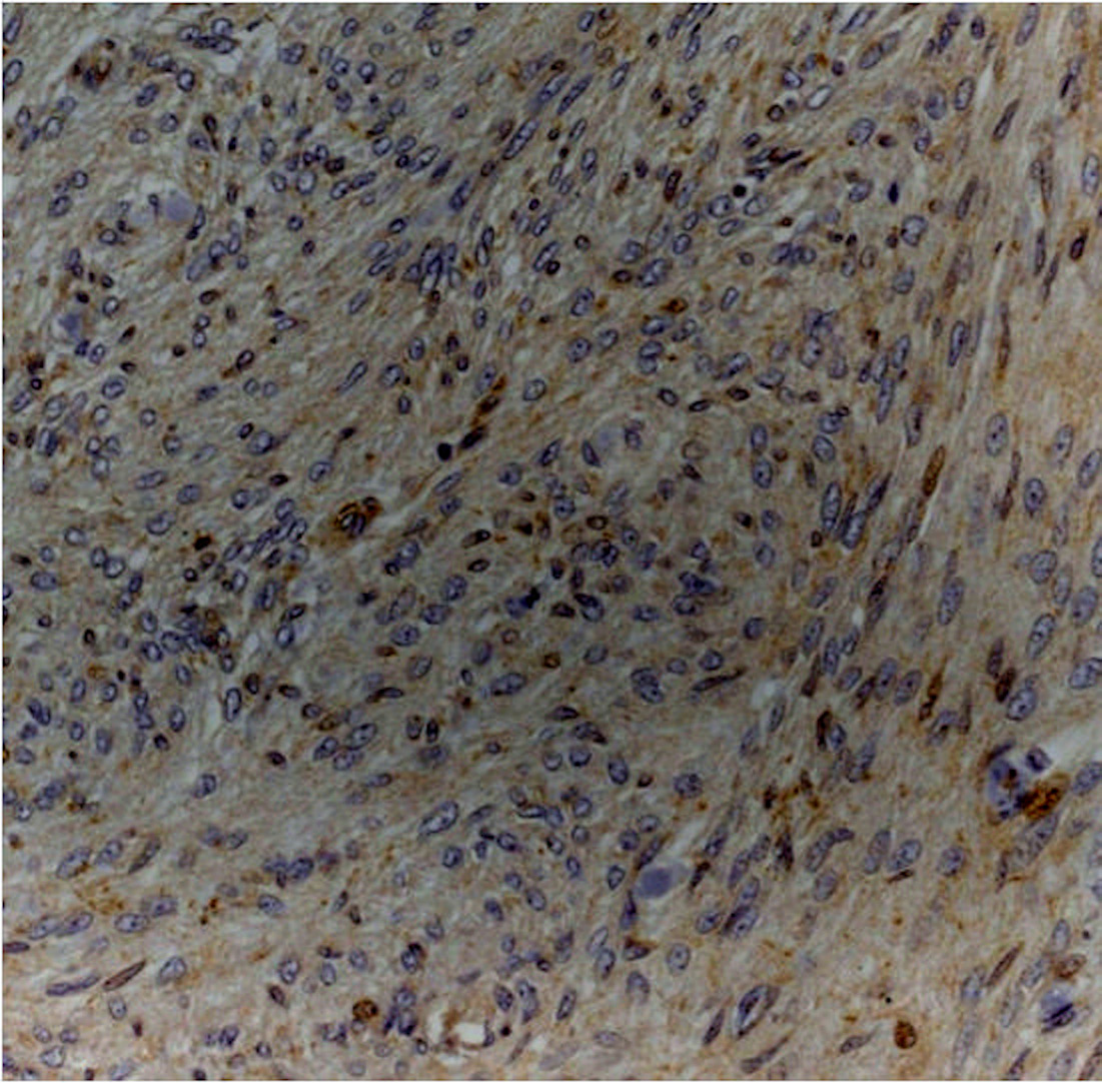
Atypical meningioma (grade II) with weak cytoplasmic staining for Dkk3 (Dkk3 stain; original magnification, x200)

Immunohistochemistry analysis showed that Dkk-3 and claudin-5 were expressed in all cases, though with different ID score and sub-cellular distribution. In particular, among grade I meningiomas, Dkk-3 had ID score 1 in 4 cases, ID score 2 in 7 cases, ID score 4 in 2 cases and ID score 8 in 2 cases (Figure 1). On the other hand, in grade II meningiomas Dkk-3 ID score was 1 in 11 cases, 2 in 3 cases and 3 in 1 case (Figure 1). In addition, while all grade I meningiomas had both cytoplasmic and nuclear staining for Dkk-3 (Figure 2), grade II tumors had only cytoplasmic staining for this protein (Figure 3). Dkk-3 immuno-expression was also found in the nuclei of the endothelial cells of some vessels in all meningiomas.

**Figure 4:**
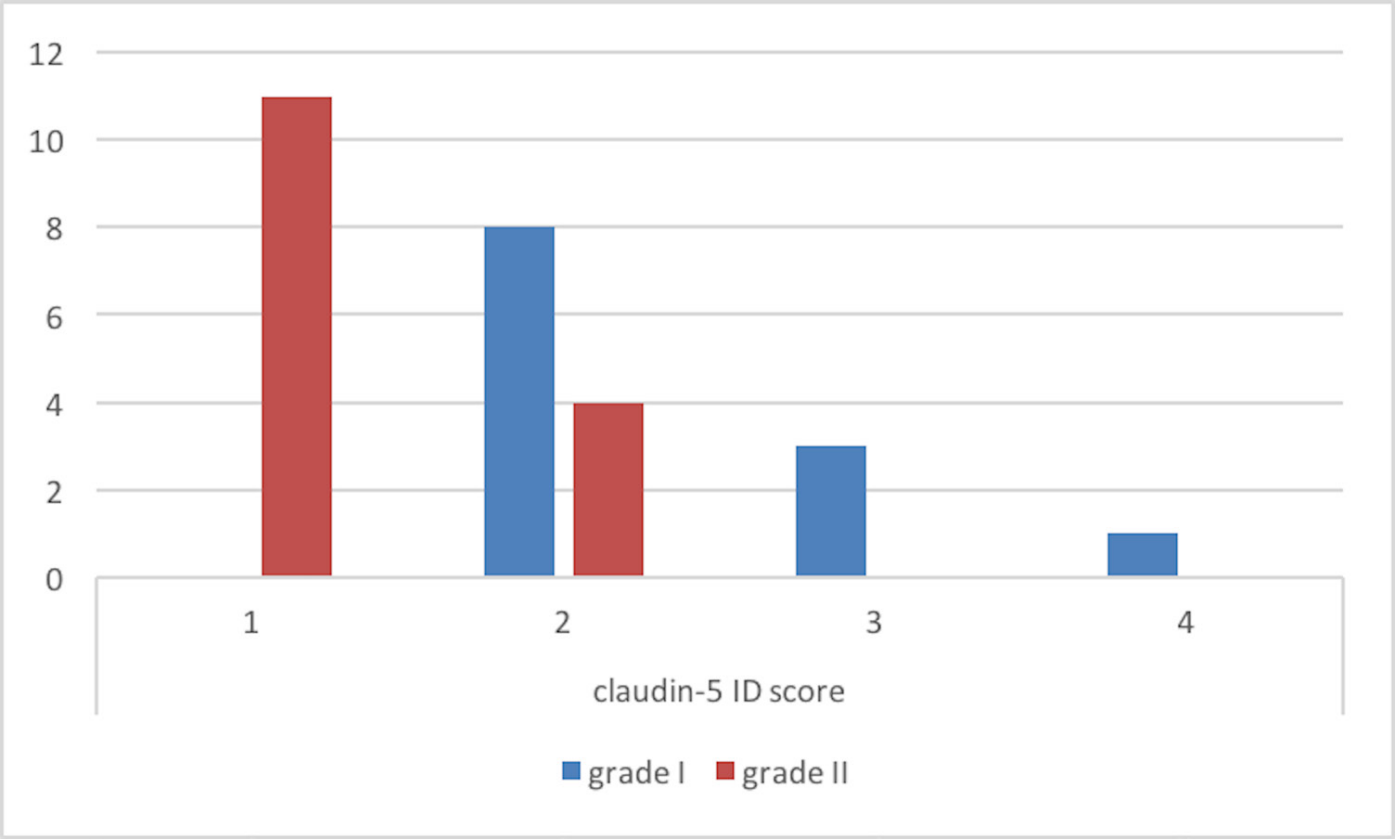
Claudin-5 immuno-expression in meningiomas

**Figure 5:**
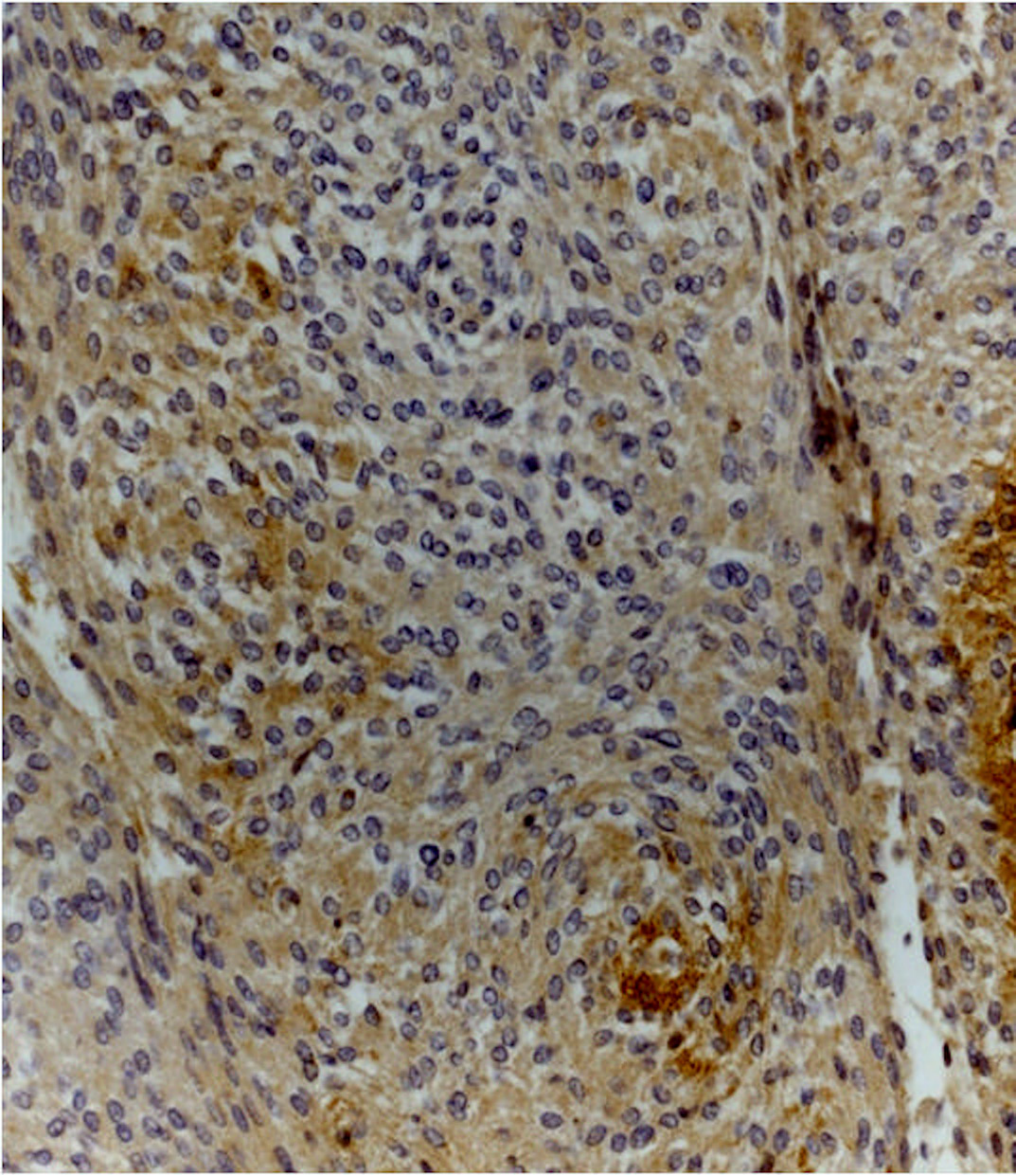
Meningothelial meningioma (grade I) showing moderate cytoplasmic staining for claudin-5 (claudin-5 stain; original magnification, x200)

ID score for claudin-5 was 2 in 8, 3 in 4 and 4 in 1 grade I meningiomas (Figure 4). On the other hand, among grade II meningiomas, 11 cases had claudin-5 ID score 1 and 4 cases had ID score 2 (Figure 4). In all meningiomas, staining was cytoplasmic (Figure 5). These results were confirmed by immunohistochemistry that revealed a slight immunoreactivity for Dkk-3 protein in meningiomas, compared with control tissues. It was also highlighted an irregular positivity of claudin-5 protein, with vessel location, due to the alteration of its function, highlighting its role in maintaining the integrity of the barrier. Chi-squared test showed that lower Dkk-3 and claudin-5 ID score were significantly associated with histological grade II (*P* = 0,0427; *P* = 0,0006). No significant correlations were observed between Dkk-3 and claudin-5 ID scores and site or Simpson grade of the tumors. A trend towards correlation was evidenced between lower Dkk-3 or claudin-5 ID scores and recurrence; however statistical significance was not reached (*P* > 0,05).

**Figure 6:**
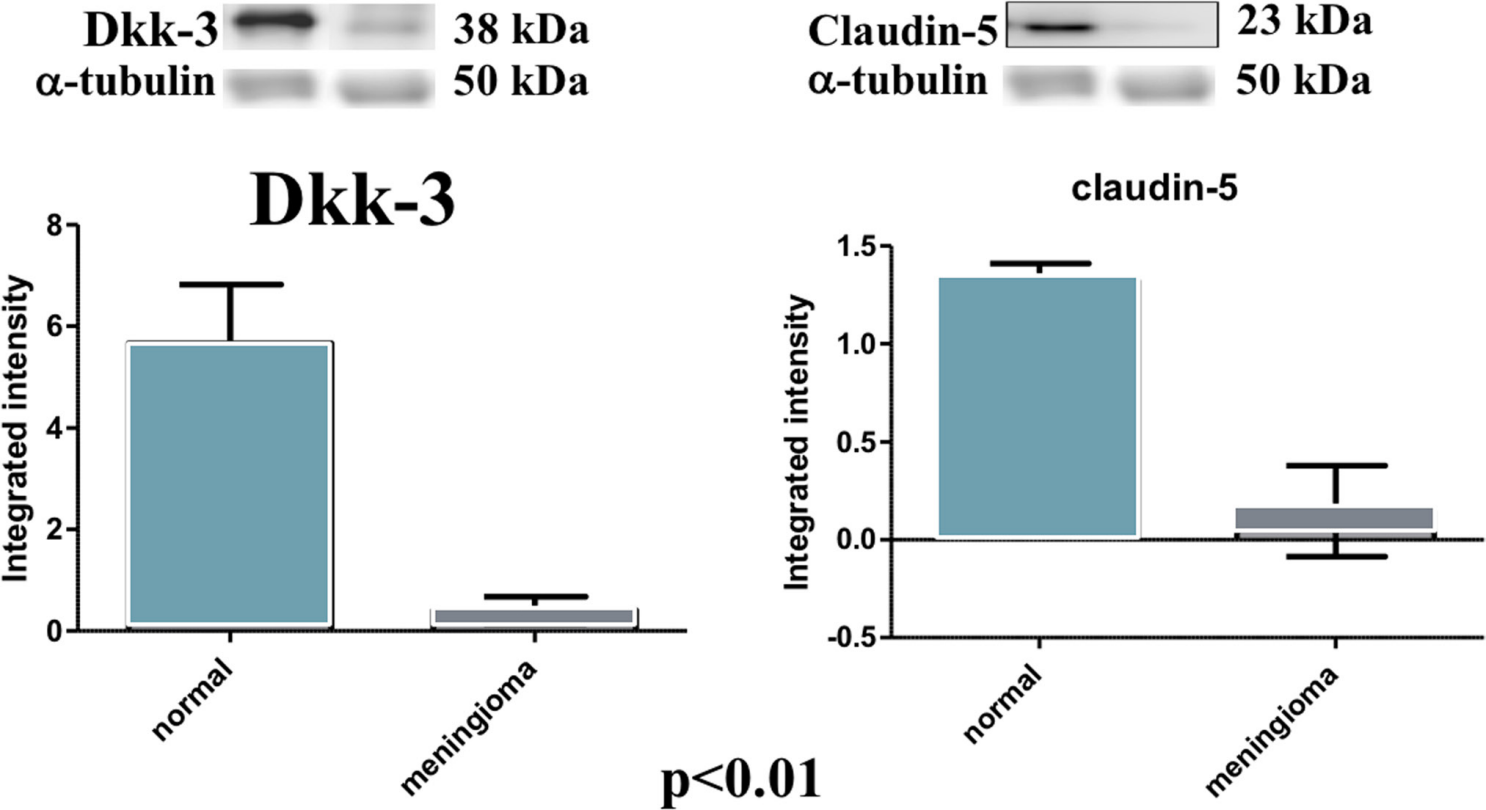
Evident decrease, at western blot analysis, of Dkk-3 and claudin-5 expression in the tissues of patients with meningiomas compared with control tissues At statistical analysis, a significant difference (*P* < 0.05) was found in the expression levels of Dkk-3 and claudin-5.

**Figure 7:**
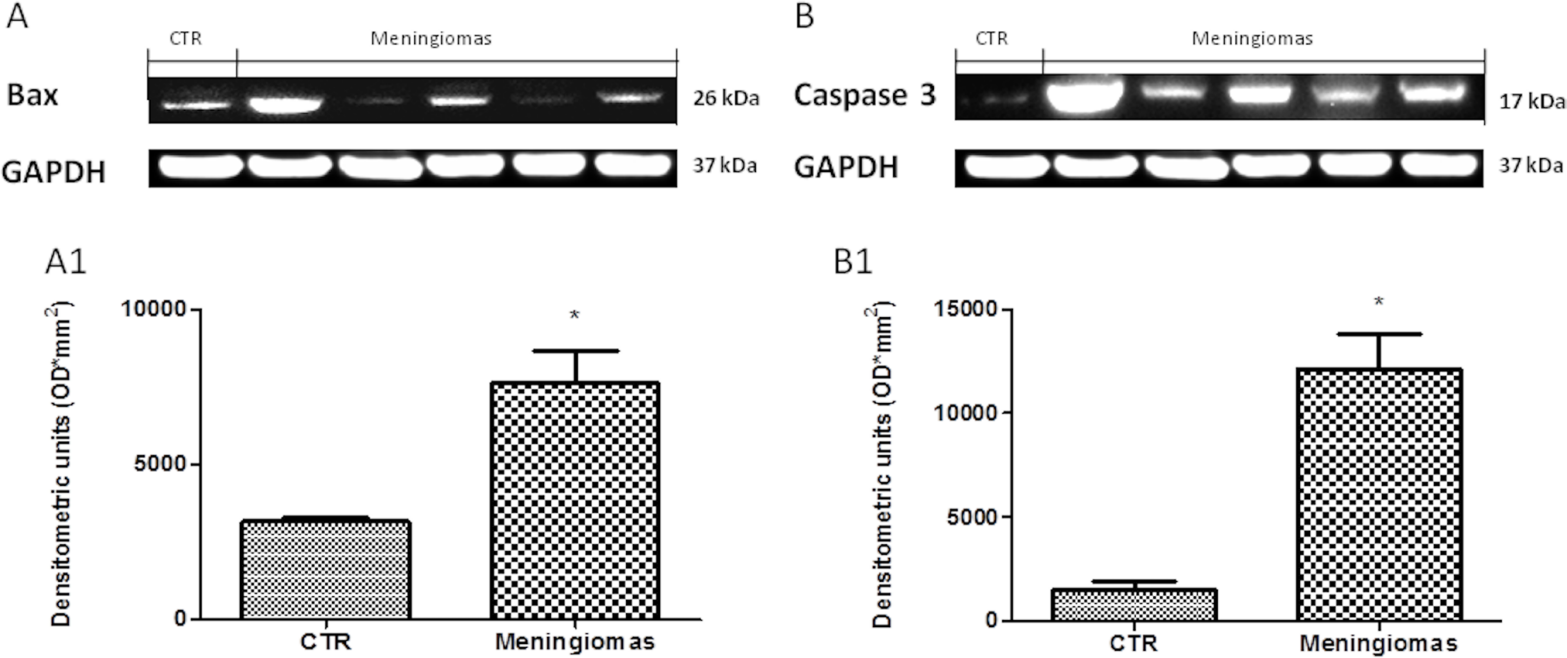
A significant increasing of pro-apoptotic proteins (Bax and Caspase 3) expression was observed in the tissue from meningiomas patients compared to control (A and B respectively, see densitometric analysis A1 and B1 respectively) Data show two representative blot from three independent experiments with similar results. Data are expressed as Mean ± SEM from *N* = 30 patients. *P* < 0.01 vs. CTR.

At western blot analysis, the data have shown a significantly reduction of Dkk-3 and claudin-5 expression in the meningioma tissue compared with control tissues (Figure 6). It has been shown that Dkk-3 has a tumor-suppressive function and a pro-apoptotic effect, inducing apoptosis through mitochondrial and Fas receptor pathways in human ovarian cancer [1]. Looking at the patients with meningiomas through western blot analysis, we evaluated the expression of Bax and Caspase 3, two important proteins on apoptosis pathways. Our result showed an upregulation of both proteins in meningiomas patient compared to control group (Figure 7) suggesting that Dkk-3 could have a pro-apoptotic function also in meningiomas.

## DISCUSSION

Meningiomas show an incidence rates of 3–4 cases per 100 000 males per year and 9–13 cases per 100 000 females per year [9]. The vast majority of meningiomas (90%) are benign, well circumscribed and characterized by a slow progression. In their progression, meningiomas can infiltrate into the surrounding bone and dura mater, and occasionally, local microinvasion into the brain may occur [1]. Finger-like extensions at the interface between tumor and normal brain might be an expression of invasion [10]. In any case, the tumor progression, although slow, cause progressive neurological deficits that can greatly affect the patient's quality of life.

Gross total resection remains the optimal objective in the treatment of all types of meningiomas [11]. However, recurrence of these lesions is often observed, and a new surgical treatment with an increased morbidity and mortality risk for the patients, could be necessary. Tenacious adhesions to vascular and eloquent structures could make impossible the total debulking of the tumor. More, rock-hard consistency and widespread adhesions to surrounding structures represent the most common troubles to total excision of these lesions. For the above-mentioned reasons, the surgical treatment and the related complications make difficult the management of such benign lesions. Many studies are now investigating clinical and molecular/genetic features of meningiomas that might contribute to better understand the behavior of the disease.

The Wingless (Wnt) signaling pathway is implicated in normal physiological processes in adult tissues, but also in embryogenesis and carcinogenesis. In human cancers, it plays an important role in uncontrolled cell growth [12–13]. The pathway is divided in two distinct branches, the canonical way (or β-catenin) and non-canonical pathway (or planar cell polarity (PCP) and Wnt-Ca2+ pathways). In the canonical pathway, the signal is transduced to β-catenin which enters into the nucleus and forms a complex with T-cell factor (TCF) to activate transcription of Wnt target genes. Non-canonical Wnt pathways are β-catenin-independent, and are often linked with the establishment of polarity and cytoskeleton-mediated processes [12]. Aberrations in Wnt signaling are responsible for the development and invasion of astrocytic brain tumors [13]. Howng et al. demonstrated that Wnts 5a, 10b and 13 are expressed in most brain tumors including meningiomas [14]. In GBM, dysregulation of Wnt signaling is mainly caused by inactivation of pathways inhibitors [15]. In a recent study RNA-binding motif protein 5 (RBM5) was demonstrated showing a suppressor role in gliomas by inhibiting Wnt/β-catenin signaling and inducing cell apoptosis [16].

Wnt/β-catenin pathway is also associated in meningioma progression through an altered expression of several of its genes. It has been reported that the mutations of adenomatous polyposis coli (*APC*) and of the E-cadherin (*CDH1*) are showed in about one-third and half of the cases [3]. Downregulation of E-cadherin expression in aggressive meningiomas had been reported in association with upregulation of β-catenin [17]. Pérez-Magán et al. [18] recently reported a gene expression profiling (GEP) signature of advanced and recurrent meningiomas, which included aberrant expression of genes of the Wnt pathway; thus, these authors found downregulation of *SFRP1*, a gene from the SFRPs (secreted frizzled-related proteins) protein family which are able to down-regulate Wnt signaling, in recurrent and atypical meningiomas. Similarly, the *BCR* gene, which typically shows lower expression in meningiomas, has also been shown to act as a negative regulator of the Wnt pathway [19].

Various extracellular Wnt antagonists can interfere with the activity of this pathway. Members of the secreted frizzled-related protein (Sfrp) family and Wnt-inhibitory factor-1 (Wif-1) directly sequester Wnt ligands, whereas Dickkopf-related proteins (Dkk) interfere with lipoprotein receptor-related protein (Lrp) and Kremen co-receptors preventing Wnt from binding a functional receptor complex.

Dkk genes comprise a gene family of four members (Dkk1-4) and a unique Dkk-3-related gene, Dkkl1 (soggy). They encode secreted glycoproteins that typically antagonize Wnt/β-catenin signaling by preventing the interaction of Wnts with the co-receptors LRP5/6 [20]. Dkks play an important role in vertebrate development, while, in the adult, are implicated in bone formation and bone disease, cancer and Alzheimer's disease. Reduced expression in immortalized cells/Dickkopf-3 (REIC/Dkk-3) was identified as a gene whose expression is reduced in a variety of human cancer cells [21–22]. The molecular mechanisms underlying the tumor-suppressing function of REIC/Dkk-3 in human glioma remain obscure. Probably it is related to REIC/Dkk-3 that regulates the growth and survival of glioma cells by caspase-dependent and -independent mechanisms, via modification of the Wnt signaling pathway. Loss of Dkk-3 expression is particularly observed in some types of cancer; particularly, in *in vitro* studies was revealed that the impact of Dkk-3 on tumor growth is probably mediated by regulation of apoptosis and accompanied by changes on cell morphology [23]. In an interesting study it was shown that down-regulation of Dkk-3 is a key event in the progression of gliomas [24]. In this study the expression of Dkk-3 was suppressed to undetectable levels in GBM and was lower in malignant glioma cell lines than in normal human astrocytes. Dkk-3 would seem to be able to stimulate caspase-dependent apoptotic mechanisms contributing to the degradation of β-catenin [24]. In another, recent, study has documented the effectiveness of Dkk-3 as a tumor-suppressor in glioma cell lines (U87**Δ**EGFR and GL261). This study showed a significant decrease of glioma cells as consequence of an up-regulation of Dkk-3 [25]. It has been showed that the overexpression of adenovirus-mediated REIC/Dkk-3 (Ad-REIC) acted via c-Jun-NH2-kinase (JNK) and c-Jun [26] to induce apoptosis in cancer cells.

Claudins, are small proteins (20-27 kDa), with a short N-terminal cytoplasmic region, two extracellular loops, and short C-terminal tail. However, the exact function of each type of claudins remains unclear. Claudins are tight junction (TJ) proteins, which along with adherens junctions and desmosomes form cellular sheets. The principal claudin in brain endothelial cells is claudin-5, but other claudins (especially claudin-1, -3 and -12) have also been detected [27]. Tumor cells frequently show abnormal TJ function as well as decreased differentiation and cell polarity. Loss of epithelial integrity with change in claudins levels and resultant increased para-cellular leakage plays a critical role in providing a space for tumor cell mobility and increased nutrients’ supply for tumor cells [28]. Claudin-5 is encoded by the *CLDN5* gene, which contains an intron and has two transcript variants [29]. This protein is located in BBB endothelial cells and represents the key factor involved in the endothelial permeability of the BBB [30]. The major role of claudin5 is selectively decreasing the permeability to ions. Downregulation of claudin-3 and claudin-5 expressions in high-grade glioma has been reported [31–32]. Downregulation of expression of claudin causes loosening of BBB tight junctions, in fact, claudin-5 can interact with claudin-3 and the selective loss of the latter during human GBM is associated with BBB breakdown [33]. This event could be associated to disturbance of TJ complex and loss of staining for claudin-5, occludin and ZO-1. More, accumulation of growth factors (vascular endothelial growth factor, or hepatocyte growth factor) as well as proinflammtory cytokines can increase the alterations in BBB permeability [34]. EscuderoEsparza et al. [35] showed, inserting claudin5 into a human vascular endothelial cell line, a significant down-regulation of motility, adhesive to matrix and angiogenic potential of vascular endothelial cells. Nitta et al. showed that claudin-5 knockout mice have an impaired BBB that was leaky to molecules up to 800 Da in size [36]. It has been hypothesized that the peritumoural brain edema in GBM is a result of the down-regulation of claudin1 and 5 and occludin expression [31].

In this study, for the first time, we have evaluated the expression of Dkk-3 and claudin-5 in a series of meningiomas. From a study of recent and relevant literature, we did not find any publication on the topic. Our data show similar findings with results published in literature concerning the expression in gliomas of these two proteins. Actually, our immunohistochemical analysis, showed reduced positivity of Dkk-3 in meningiomas of grade I, even more accentuated in meningiomas grade II. The interesting finding was the positivity of Dkk-3 in the endothelial cells of tumor vessels. According to Untergasser's observations, probably various proteins such as VEGF, fibroblast growth factors (FGF), granulocyte macrophage colony-stimulating factor (GM-CSF), produced by neoplastic cells, could increase the expression of Dkk-3 [37].

Interestingly, in a similar manner, also the values of claudin-5 are poorly expressed in both sets of meningiomas. The altered expression of claudin-5, probably related to the overproduction of cytokines of tumor cells, could cause an alteration of the adjustment of the BBB with an increase of the permeability favoring, thus, cell migration and edema formation. It must be emphasized, although our data are preliminary, as cancer cells are able to alter, in a synergistic way, the production of these proteins by creating a suitable environment for their proliferation.

Despite the majority of meningiomas are defined as benign lesions, they show a clear tendency to recurrence. To date, the effective treatment for meningiomas remains surgery, even if tenacious adhesion to eloquent anatomical structures and/or important vessels or nerves, cannot often allow the total removal of the lesion. In the light of these observations, the attention was focused on two possible targets: Dkk-3 and claudin-5. The Dkk-3, that is able to regulate the WNT pathway, while the claudin-5 appears to interfere in maintaining the integrity of BBB permeability. By molecular analysis, our findings suggest a key role of Dkk-3 protein in meningiomas, as a potential tumor suppressor, evidenced by its reduced expression in tumor tissues. In addition, the down-regulation of claudin-5 observed in our study confirms the protective role of BBB to maintain its integrity. Moreover, it has been shown that Dkk-3 is able to modulate apoptosis in many tumorigenesis process such as in ovaric cancer [1]; our work clearly demonstrated that Dkk-3 in meningiomas had pro-apoptotic effects up-regulating both intrinsic and extrinsic apoptotic pathways. The data obtained in this study demonstrate that these proteins are poorly expressed and that these values are even more evident in the series of grade II meningiomas. It is expected that the restoration of the levels of these proteins could induce a slowdown in tumor progression. Obviously, we know that the data were obtained from a small number of cases and new studies will be needed to confirm our hypothesis. However, a more deepen identification of the role of these proteins might suggest interesting new molecular targets for future therapeutic protocols.

## MATERIALS AND METHODS

Our study includes 30 cases of meningiomas of different histological grading divided into two series. By reviewing patient records and contacting patients, families, and referring physicians, the following information was gathered: age, sex, tumor location, clinical presentation, and surgical data. The extent of surgical resection was evaluated by the Simpson grading system [38]. This scale divides the extent of tumor resection in 5 grades:

Grade 1: complete removal

Grade 2: complete removal with coagulation of dural attachment

Grade 3: complete removal, without coagulation of dural attachment or resection of involved sinus or hyperostotic bone

Grade 4: subtotal resection

Grade 5: decompression-biopsy

The molecular analysis were performed on frozen sections of meningioma specimens, by means of Western Blot method, in order to study the specific antibody of interest, like as Dkk-3 and claudin-5. Patient informed consent for the use of tissue samples obtained at surgery was also acquired. As control tissue, healthy dura mater from patients operated on decompressive craniectomy for severe head trauma was used. It was also carried out statistical analysis.

### Histopathological analysis

The histopathological analyses were performed in the Neuropathology Laboratory of the department of Human Pathology at the University of Messina, Italy. Each surgical specimen had been formalin fixed for 24 hours at room temperature, paraffin-embedded at 56°C, and cut into parallel 4mμ-thick sections for histological evaluation with haematoxylin and eosin stain (H&E) and for the immunohistochemical analyses against Dkk-3 and claudin-5. Histological diagnosis (including histotype and histological grade) of each case was established according to recent WHO classification [2].

### Immunohistochemistry

The intrinsic endogenous peroxidase activity was blocked with 0.1% H_2_O_2_ in methanol for 20 min.; then, normal sheep serum was applied for 30 min. to prevent unspecific adherence of serum proteins. Dkk-3 and claudin-5 antigens were unmasked by microwave oven pre-treatment in 10 mM, pH 6.0 sodium citrate buffer for 3 cycles x 5 min. Consecutive sections were successively incubated with the primary antibodies against Dkk-3 (Abcam, Cambridge, UK; working dilution, 1:100) and claudin-5 (Santa Cruz Biotechnology, Heidelberg, Germany; working dilution, 1:100) by using Dako Autostainer. The bound primary antibodies were visualized by using envision system (Dako Cytomation, Glostrup, Denmark) according to the manufacturer's instructions. To reveal the immunostaining, the sections were incubated in darkness for 10 min. with 3-3′ diaminobenzidine tetra hydrochloride (Sigma Chemical Co., St. Louis, MO, USA), in the amount of 100 mg in 200 ml 0.03 % hydrogen peroxide in phosphate-buffered saline solution (PBS). Nuclear counterstaining was performed by Mayer's haemalum. Immunohistochemical expression of Dkk-3 and claudin-5 was assessed semi-quantitatively, by using the so-called ID score. In detail, in all cases the intensity of staining (IS) was classified as 0 (absent), 1 (weak), 2 (moderate), 3 (strong). The ASP, which reflected the percentage of positive cells, was rated as follows 0 (less than 5% of stained cells), 1 (5–25% stained cells), 2 (26–50% stained cells), 3 (51–75% stained cells), 4 (> 75% stained cells). Then, for each case ID score was calculated by multiplying the values of IS and ASP.

### Western blot analysis

Total cellular proteins of meningioma tissue were extracted in a lysis buffer composed by 25 mMTris–HCl pH 7.4, 1.0 mM ethylene glycol tetraacetic acid (EGTA), 1.0 mMethylenediaminetetraacetic acid (EDTA), 0.5 mM phenyl methylsulphonyl fluoride, also with protease and phosphatase inhibitors [100 mM Na3VO4, aprotinin, leupeptin, pepstatin (10 μg/ml each)]. Then cell lysate obtained was centrifuged at 13000 rpm for 15 minutes and the supernatant was used for determination of protein concentration by Bio-Rad protein assay (Bio-Rad, Richmond, CA, USA) and diluted with Laemmli buffer. Meningioma specimens were denatured in buffer constituted by 62 mMTris pH 6.8, 10% glycerol, 2% SDS, 5% b-mercaptoethanol, 0.003% bromophenol blue, separated by electrophoresis on SDS polyacrylamide gel, and then transferred on to nitrocellulose membrane at 200 mA for 1 h. The membranes were blocked with 5% non-fat dry milk in TBS-0.1% Tween-20 for 1 h at room temperature, washed three times for 10 min each in TBS-0.1% Tween-20 and incubated with a primary antibody for Dkk-3 (Abcam, Cambridge, UK), claudin-5 (1:500, Santa Cruz Biotechnology, Heidelberg, Germany), Bax (1:500 Santa Cruz Biotechnology) and Caspase 3 (1:500 Santa Cruz Biotechnology), overnight at 4°C. The day after the membranes were incubated with a specific peroxidase-conjugated secondary antibody (Pierce, Cramlington, UK) for 1 h at room temperature and were analyzed by the enhanced chemiluminescence (KPL, USA). Protein signals were quantified by scanning densitometry using a bio-image analysis system (Bio-Profil, Milan, Italy) and the results were expressed as relative integrated intensity compared to controls. β-actin (Cell Signaling Technology, Beverly, MA, USA) and GAPDH (1:500, Santa Cruz Biotechnology, Heidelberg, Germany) was used to confirm equal protein loading and blotting.

### Statistical analysis

All values, in the figures, were evaluated as mean ± SEM. The results were examined by one-way analysis of variance followed by a Bonferroni post-hoc test for multiple comparisons; *p* values < 0.05 were considered statistically significant. Chi-squared test was used to analyze the correlations between Dkk-3 or claudin ID scores and the histological grade, site, Simpson grade and recurrences of meningiomas.

## Abbreviations

WHO: World Health Organization
BBB: blood-brain barrier
TJ: tight junction
MRI: magnetic resonance imaging
GBM: glioblastoma multiforme
ID: intensity distribution
IS: intensity of staining
ASP: area of staining positivity
SEM: standard error of the mean
